# Association between acetaminophen administration and clinical outcomes in patients with sepsis admitted to the ICU: a retrospective cohort study

**DOI:** 10.3389/fmed.2024.1346855

**Published:** 2024-01-31

**Authors:** Shilin Sun, Han Liu, Qun Liang, Yang Yang, Xuedan Cao, Boyang Zheng

**Affiliations:** ^1^Heilongjiang University of Chinese Medicine, Harbin, China; ^2^Institute for Global Health, University College London, London, United Kingdom; ^3^The First Affiliated Hospital of Heilongjiang University of Chinese Medicine, Harbin, China

**Keywords:** acetaminophen, sepsis, mortality, critical care, MIMIC-IV

## Abstract

**Background:**

Sepsis, affecting over 30 million people worldwide each year, is a key mortality risk factor in critically ill patients. There are significant regional discrepancies in its impact. Acetaminophen, a common over-the-counter drug, is often administered to control fever in suspected infection cases in intensive care units (ICUs). It is considered generally safe when used at therapeutic levels. Despite its widespread use, there’s inconsistent research regarding its efficacy in sepsis management, which creates uncertainties for ICU doctors about its possible advantages or harm. To address this, we undertook a retrospective cohort study utilizing the MIMIC-IV database to examine the correlation between acetaminophen use and clinical outcomes in septic patients admitted to the ICU.

**Methods:**

We gathered pertinent data on sepsis patients from the MIMIC-IV database. We used propensity score matching (PSM) to pair acetaminophen-treated patients with those who were not treated. We then used Cox Proportional Hazards models to examine the relationships between acetaminophen use and factors such as in-hospital mortality, 30-day mortality, hospital stay duration, and ICU stay length.

**Results:**

The data analysis involved 22,633 sepsis patients. Post PSM, a total of 15,843 patients were matched; each patient not receiving acetaminophen treatment was paired with two patients who received it. There was a correlation between acetaminophen and a lower in-hospital mortality rate (HR 0.443; 95% CI 0.371–0.530; *p* < 0.001) along with 30-day mortality rate (HR 0.497; 95% CI 0.424–0.583; *p* < 0.001). Additionally, it correlated with a decrease in the duration of hospitalization [8.4 (5.0, 14.8) vs. 9.0 (5.1, 16.0), *p* < 0.001] and a shorter ICU stay [2.8 (1.5, 6.0) vs. 3.1 (1.7, 6.5); *p* < 0.05].

**Conclusion:**

The use of acetaminophen may lower short-term mortality in critically ill patients with sepsis. To confirm this correlation, future research should involve multicenter randomized controlled trials.

## 1 Introduction

Sepsis is a life-threatening condition arising from an abnormal response to infection, which results in organ dysfunction. It remains a significant cause of death among critically ill patients (1, 2). Even though sepsis-related mortality has reduced recently, it still impacts over 30 million people every year, potentially leading to 6 million deaths (3, 4). Alarmingly, remarkable disparities exist between regions. Patients in low to middle-income countries face higher mortality rates from sepsis than those in developed countries (5, 6). Consequently, sepsis treatment and management persist to be significant obstacles.

Acetaminophen, also known as an over-the-counter medication, is deemed safe at therapeutic doses. It exhibits pain-relieving and fever-reducing properties similar to aspirin (7, 8). Nowadays, it is standard in the intensive care unit (ICU) to use acetaminophen to reduce body temperature for patients presenting with fever and potential infections (9, 10). Most patients diagnosed with sepsis show symptoms of fever (11, 12). In severe sepsis cases, hemolysis occurs, leading to the production of free hemoglobin, reactive oxygen species, and lipid peroxidation, which ultimately cause cell damage (13, 14). Study outcomes have demonstrated that acetaminophen reduces free radicals in iron protoporphyrin-free hemoglobin and hinders lipid peroxidation (15, 16). Thus, employing acetaminophen may be beneficial to sepsis patients. However, its use in sepsis treatment does not have universal agreement within the scholarly community. Some experts champion the idea of reducing body temperature, identifying fever as a harmful factor (17). Conversely, there’s a belief among others that fever during an infection can enhance survival (18, 19). Moreover, studies indicate no impact on the number of ICU-free days when acetaminophen is administered early to treat fever resulting from a probable infection (20).

Due to the lack of high-level evidence, ICU physicians currently face uncertainty regarding the benefits, effectiveness, or potential harm of acetaminophen treatment for fever in cases of sepsis (21). To address this uncertainty, we conducted a retrospective cohort study based on the MIMIC-IV database. The aim was to assess the association between acetaminophen use and in-hospital mortality, 30-day mortality, length of hospital stay, and ICU stay in sepsis patients. To be more specific, our hypothesis posits that compared to patients not using acetaminophen, the use of acetaminophen can lower short-term mortality.

## 2 Materials and methods

We utilized Navicat Premium v16.1.7 to gather data from the MIMIC-IV database v2.2, specifically focusing on sepsis patients who either did or did not use acetaminophen. This publicly accessible database provides real-world data on more than 73,000 patients who were admitted to the ICU at Beth Israel Deaconess Medical Center between 2008 and 2019 (22). Author Shilin Sun secured authorization to utilize this database (Certification Number: 12281929). All reporting in this study adheres to the guidelines stipulated by the Strengthening the Reporting of Observational Studies in Epidemiology (23).

### 2.1 Study population

We undertook a retrospective analysis of sepsis patients, setting these inclusion criteria: (1) patients must be at least 18 years old, and (2) patients must meet Sepsis-3 criteria — i.e., have a Sequential Organ Failure Assessment (SOFA) score of two or more due to a confirmed or suspected infection (1, 24). We identified infections by referencing International Classification of Diseases (ICD-9 and ICD-10) codes (25, 26). Every patient started with a default SOFA score of zero (27).

The exclusion criteria included: (1) patients younger than 18 years old, and (2) for patients with multiple ICU visits, only data from their first ICU admission were considered (28).

### 2.2 Acetaminophen use

The researchers examined the use of acetaminophen among patients within the first 48 h of ICU admission, using data extracted from the MIMIC-IV database (29).

### 2.3 Covariates

We used a predetermined set of covariates based on well-known predictors of sepsis outcomes (28, 30, 31). These factors included heart rate, mean arterial pressure (MAP), temperature, respiratory rate, SPO_2_, PaO_2_/FiO_2_, glucose levels, white blood cell (WBC) count, serum creatinine (SCr) levels, hemoglobin levels, platelet count, alanine aminotransferase (ALT), aspartate aminotransferase (AST), alkaline phosphatase (ALP), bilirubin, SOFA score, simplified acute physiology score (SAPS) II, infection site, and several comorbidities, like myocardial infarction, congestive heart failure, dementia, cerebrovascular disease, chronic pulmonary disease, mild liver disease, renal disease. Moreover, the use of medication such as statins, aspirin, vasopressin, continuous renal replacement therapy (CRRT) and acetaminophen route was considered. Important information from hospital admission records, including demographic characteristics, marital status, insurance details, and admission type, were also factored in. These variables comprehensively cover patient health behaviors, potentially revealing confounding effects in those treated with acetaminophen (32).

### 2.4 Outcome

This study primarily focuses on in-hospital mortality, with additional outcomes being 30-day mortality the duration of hospital and ICU stays.

### 2.5 Statistical analysis

Variables of continuous nature with normal distribution were expressed as mean ± standard deviation, and an independent-samples *t*-test was used for group comparisons. On the other hand, skewed continuous variables were shown as median (IQR) and compared using the Mann-Whitney U test between groups. Categorical variables were represented by numbers and percentages, with comparisons between groups performed using the chi-squared test or Fisher’s exact test, as necessary.

In our research, we applied propensity score matching (PSM) to tackle confounding factors in the original group. This required performing a greedy nearest neighbor match with a 0.2 standard deviation caliper of the logit for the prospective propensity score (33). We employed k-nearest neighbor imputation (KNN) with a k value of 10 for imputing the matching baseline variables (34). We matched patients at a 1:2 ratio, pairing each non-acetaminophen-treated patient within 48 h of ICU admission with two treated patients. To assess the PSM’s effectiveness in reducing differences between the groups, we calculated the standardized mean difference (SMD).

We used a multivariate Cox regression model to adjust for confounding variables. These variables were selected based on the results of a univariate analysis with a *p*-value less than 0.05 and potential confounders recognized by our team’s clinical expertise. This method was applied to estimate the correlation between acetaminophen use and mortality risk (35, 36).

In our subgroup analysis, we investigated how factors such as age, sex, ethnicity, marital status, insurance, admission type, infection site, comorbidities, medication history, and intervention usage might affect the correlation between acetaminophen use and in-hospital mortality rates.

The statistical analyses were completed using IBM SPSS Statistics version 26.0 and R 4.2.2 software. A *p*-value below 0.05 was deemed statistically significant.

## 3 Results

### 3.1 Population

**FIGURE 1:**
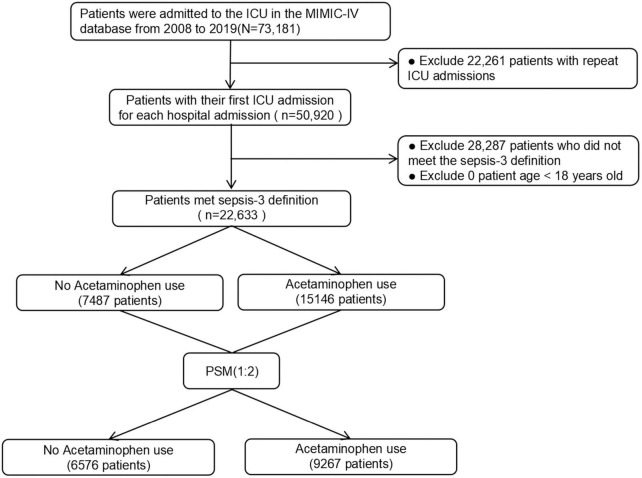
The flow chart of the study.

The study incorporated 22,633 patients diagnosed with sepsis per the Sepsis-3 definition. Among these, 15,146 (66.9%) were recognized as acetaminophen users. The patient selection process is visually represented in Figure 1.

Table 1 illustrates notable discrepancies in various foundational characteristics between the two patient groups from the original sample. These include differences in admission type, body temperature, respiratory rate, SPO_2_, PaO_2_/FiO_2_, glucose levels, SCr, ALT, AST, ALP, bilirubin, SOFA score, SAPS II score, infection site, cerebrovascular disease presence, medication usage, and interventions.

**TABLE 1:** Baseline characteristics between groups before and after PSM.

Variables	Before matching				After matching			
	**Total**	**Acetaminophen** **use**	**No acetaminophen** **use**	**SMD^Δ^**	**Total**	**Acetaminophen** **use**	**No acetaminophen** **use**	**SMD^Δ^**
*n* (%)	22,633	15,146 (66.9)	7,487 (33.1)		15,843	9,267	6,576	
Age (years)^a^		65.05 ± 16.35	65.12 ± 16.35	0.004		65.65 ± 16.80	65.67 ± 16.31	-0.021
Female, *n* (%)	9,556	6,340 (41.9)	3,216 (43.0)	0.022	6,975	4,122 (44.5)	2,853 (43.4)	-0.028
Ethnicity, white, *n* (%)	15,162	10,280 (67.9)	4,882 (65.2)	-0.056	10,542	6,202 (66.9)	4,340 (66.0)	-0.019
**Marital, status, *n* (%)**								
Married	10,122	6,995 (46.2)	3,127 (41.8)	-0.09	6,687	3,903 (42.1)	2,784 (42.3)	0.015
Single/divorced	7,457	4,859 (32.1)	2,598 (34.7)	0.055	5,415	3,157 (34.1)	2,258 (34.3)	0.002
Other	5,054	3,292 (21.7)	1,762 (23.5)	0.042	3,741	2,207 (23.8)	1,534 (23.3)	-0.02
**Insurance, *n* (%)**								
Medicaid	1,629	993 (6.6)	636 (8.5)	0.07	1,187	663 (7.2)	524 (8.0)	0.014
Medicare	10,475	6,896 (45.5)	3,579 (47.8)	0.045	7,696	4,495 (48.5)	3,201 (48.7)	-0.014
Other	10,529	7,257 (47.9)	3,272 (43.7)	-0.085	6,960	4,109 (44.3)	2,851 (43.4)	0.006
**Admission type, *n* (%)**								
Elective	5,524	4,514 (29.8)	1,010 (13.5)	-0.478	2,527	1,561 (16.8)	966 (14.7)	0.012
Emergency	17,109	10,632 (70.2)	6,477 (86.5)	0.478	13,316	7,706 (83.2)	5,610 (85.3)	-0.012
**Vital signs**								
Heart rate (BPM)^a^	86.80 ± 18.98	86.50 ± 15.46	87.39 ± 16.97	0.052	87.12 ± 16.86	86.91 ± 16.23	87.12 ± 16.86	0.004
MAP (mmHg)^a^	79.59 ± 10.33	79.46 ± 9.87	79.84 ± 11.20	0.034	80.00 ± 11.07	80.30 ± 10.45	80.00 ± 11.07	-0.029
Temperature (°C)^b^	37.39 (37.00, 37.90)	37.44 (37.06, 38.10)	37.22 (36.94, 37.67)	-0.485	37.33 (37.00, 37.89)	37.33 (37.00, 37.89)	37.28 (36.94, 37.72)	-0.069
Respiratory rate (BPM)^a^	19.62 ± 4.06	19.40 ± 3.91	20.07 ± 4.31	0.154	19.96 ± 4.22	19.80 ± 4.04	19.95 ± 4.22	0.008
SPO_2_ (%)^b^	97.24 (95.79, 98.52)	97.34 (95.95, 98.53)	97.02 (95.40, 98.52)	-0.154	97.07 (95.64, 98.41)	97.07 (95.64, 98.40)	97.04 (95.48, 98.53)	-0.018
PaO_2_/FiO_2_^b^	257 (203, 315)	261 (207, 318)	249 (192, 307)	-0.132	256 (202, 309)	256 (202, 309)	252 (196, 308)	-0.012
**Laboratory tests**								
Glucose (mg/dL)^b^	131 (114, 159)	130 (115, 153)	137 (112, 174)	0.183	133 (113, 162)	133 (113, 161)	135 (111, 171)	0.034
WBC (x10^9^)^b^	12 (9, 16)	12 (9, 16)	12 (8, 16)	-0.014	12 (9, 16)	12 (9, 16)	12 (8, 16)	-0.002
SCr (mg/dL)^b^	1.05 (0.75, 1.60)	1.00 (0.75, 1.40)	1.20 (0.80, 2.00)	0.238	1.05 (0.75, 1.60)	1.05 (0.75, 1.60)	1.15 (0.80, 1.90)	0.036
Hemoglobin (g/L)^a^	10.69 ± 2.01	10.72 ± 1.95	10.64 ± 2.14	-0.035	10.69 ± 2.12	10.76 ± 2.05	10.69 ± 2.12	-0.017
Platelets (x10^12^)^b^	180 (129, 246)	179 (133, 240)	184 (120, 257)	0.026	190 (135, 257)	190 (135, 257)	189 (126, 260)	-0.038
ALT (IU/L)^b^	31 (20, 60)	29 (20, 50)	37 (21, 93)	0.179	31 (20, 58)	31 (20, 58)	34 (20, 77)	0.056
AST (IU/L)^b^	46 (31, 90)	44 (31, 75)	55 (31, 140)	0.193	46 (30, 85)	46 (30, 85)	50 (29, 114)	0.055
ALP (IU/L)^b^	75 (60, 105)	71 (58, 95)	86 (66, 125)	0.218	78 (62, 108)	78 (62, 108)	83 (64, 119)	0.028
Bilirubin (μmol/L)^b^	0.73 (0.50, 1.15)	0.70 (0.50, 1.00)	0.80 (0.50, 1.80)	0.252	0.70 (0.50, 1.10)	0.70 (0.50, 1.10)	0.76 (0.50, 1.45)	0.063
**Severity of illness**								
SOFA score^b^	3.00 (2.00, 4.00)	3.00 (2.00, 4.00)	3.00 (2.00, 5.00)	0.239	3.00 (2.00, 4.00)	3.00 (2.00, 4.00)	3.00 (2.00, 5.00)	0.072
SAPS II score^a^	39.58 ± 14.49	37.87 ± 13.58	43.01 ± 15.64	0.329	41.60 ± 14.80	39.53 ± 14.19	41.60 ± 14.80	0.067
**Infection site (%)**								
Respiratory system	10,544	6,527 (43.1)	4,017 (53.7)	0.212	8,268	4,779 (51.6)	3,489 (53.1)	-0.004
Digestive system	3,479	2,197 (14.5)	1,282 (17.1)	0.069	2,480	1,403 (15.1)	1,077 (16.4)	0.013
Urogenital system	3,728	2,669 (17.6)	1,059 (14.1)	-0.1	2,484	1,505 (16.2)	979 (14.9)	-0.017
Cardiovascular system	1,210	1,045 (6.9)	165 (2.2)	-0.32	424	264 (2.8)	160 (2.4)	0.009
Other	3,672	2,708 (17.9)	964 (12.9)	-0.149	2,187	1,316 (14.2)	871 (13.2)	0.006
**Comorbidity disease, *n* (%)**								
Myocardial infarct	3,792	2,574 (17.0)	1,218 (16.3)	-0.02	2,609	1,525 (16.5)	1,084 (16.5)	0.004
Congestive heart failure	6,356	4,108 (27.1)	2,248 (30.0)	0.063	4,755	2,734 (29.5)	2,021 (30.7)	0.001
Dementia	1,034	693 (4.6)	341 (4.6)	-0.001	826	501 (5.4)	325 (4.9)	-0.019
Cerebrovascular disease	3,169	2,285 (15.1)	884 (11.8)	-0.102	2,171	1,344 (14.5)	827 (12.6)	-0.036
Chronic pulmonary disease	5,820	3,709 (24.5)	2,111 (28.2)	0.082	4,374	2,523 (27.2)	1,851 (28.1)	-0.001
Mild liver disease	3,249	1,403 (9.3)	1,846 (24.7)	0.357	2,499	1,229 (13.3)	1,270 (19.3)	0.08
Renal disease	4,791	2,969 (19.6)	1,822 (24.3)	0.11	3,702	2,119 (22.9)	1,583 (24.1)	-0.006
**Medications use, *n* (%)**								
Aspirin	7,431	5,953 (39.3)	1,478 (19.7)	-0.491	3,795	2,382 (25.7)	1,413 (21.5)	-0.013
Statin	7,229	5,791 (38.2)	1,438 (19.2)	-0.483	3,837	2,439 (26.3)	1,398 (21.3)	-0.033
**Interventions, *n* (%)**								
Vasopressin	2,422	1,314 (8.7)	1,108 (14.8)	0.172	1,738	935 (10.1)	803 (12.2)	0.036
CRRT	1,199	525 (3.5)	674 (9.0)	0.193	880	432 (4.7)	448 (6.8)	0.046
**Acetaminophen route, *n* (%)**								
PO/NG	11,007	11,007 (72.7)	–	–	7,999	7,999 (86.3)	–	–
IV	3,160	3,160 (20.9)	–	–	1,258	1,258 (13.6)	–	–
PR	979	979 (6.5)	–	–	10	10 (0.1)	–	–

^a^Descriptive statistics were calculated using the mean (standard deviation), mean ± SD.

^b^Descriptive statistics were calculated using the median (interquartile range, IQR),[median (IQR)].

^Δ^Standardized mean difference.

**FIGURE 2:**
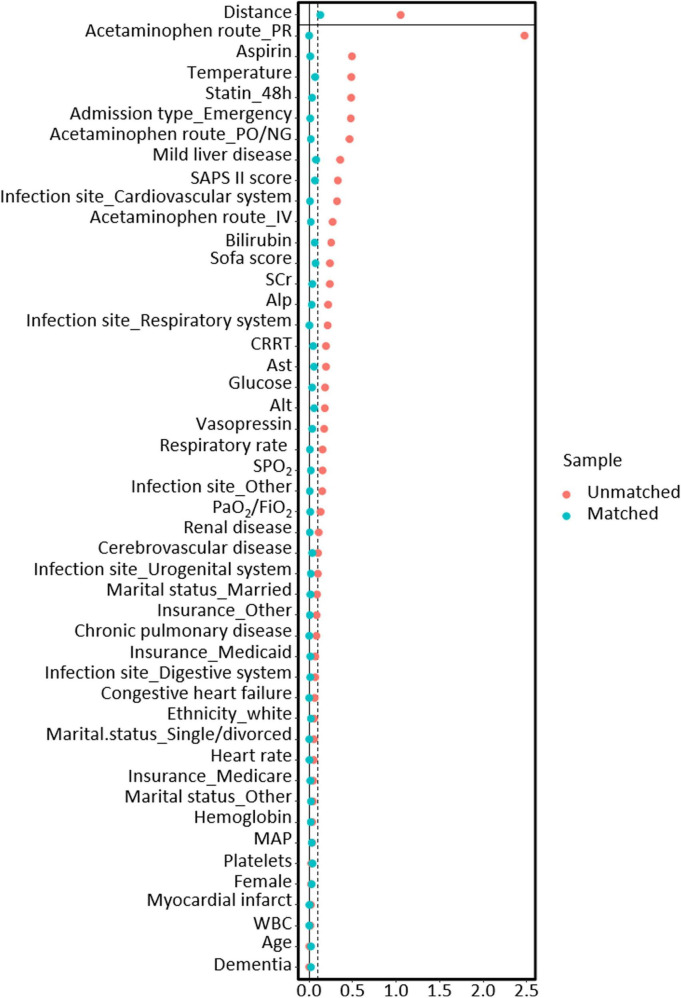
The relationship between SMD and all covariates before and after propensity score matching models.

After PSM, 9,267 patients treated with acetaminophen were matched with 6,576 patients who did not receive acetaminophen treatment. After matching, there was a good balance in baseline characteristics between the two groups, with all variables having a SMD of less than 10% (Figure 2).

### 3.2 Association between acetaminophen utilization and clinical outcomes

In the initial group, we noticed a link between acetaminophen use and a lower in-hospital death rate (HR 0.432; 95% CI 0.405–0.462; *p* < 0.001). This correlation stayed statistically significant even after adjusting for possible confounding factors (HR 0.512; 95% CI 0.448–0.585; *p* < 0.001). We also evaluated the effect of acetaminophen use on 30-day mortality, overall hospital stay duration, and length of ICU stay. The data showed that usage of acetaminophen led to a lower 30-day death rate (HR 0.582; 95% CI 0.518–0.655; *p* < 0.001), shorter hospital stay [7.9 (5.0, 13.8) vs. 9.1 (5.1, 16.6), *p* < 0.001], and shorter ICU stay [2.6 (1.4, 5.3) vs. 3.2 (1.7, 6.7); *p* < 0.001] (Table 2).

**TABLE 2:** Association between acetaminophen use and clinical outcomes in sepsis patients.

	No acetaminophen use	Acetaminophen use	*P*-value	HR	Lower 95% CI	Upper 95% CI
Pre-matched cohort	*n* = 7,487	*n* = 15,146				
**Primary outcome**						
In-hospital mortality, *n* (%)^a^	1,767 (23.6)	1,684 (11.1)	< 0.001	0.512	0.448	0.585
**Secondary outcomes**						
30-day mortality, *n* (%)^a^	2,110 (28.2)	2,145 (14.2)	< 0.001	0.582	0.518	0.655
Length of hospital stay (day), [median (IQR)]	9.1 (5.1, 16.6)	7.9 (5.0, 13.8)	< 0.001	1.762	1.398	2.125
Length of ICU stay (day), [median (IQR)]	3.2 (1.7, 6.7)	2.6 (1.4, 5.3)	< 0.001	0.631	0.449	0.813
Post-matched cohort	*n* = 6,576	*n* = 9,267				
**Primary outcome**						
In-hospital mortality, *n* (%)^a^	1,361 (20.7)	1,271 (13.7)	< 0.001	0.443	0.371	0.530
**Secondary outcomes**						
30-day mortality, *n* (%)^a^	1,655 (25.2)	1,634 (17.6)	< 0.001	0.497	0.424	0.583
Length of hospital stay (day), [median (IQR)]	9.0 (5.1, 16.0)	8.4 (5.0, 14.8)	< 0.001	1.024	0.617	1.431
Length of ICU stay (day), [median (IQR)]	3.1 (1.7, 6.5)	2.8 (1.5, 6.0)	0.036	0.218	0.015	0.422

^a^Adjusted for all the factors (acetaminophen use, age, sex, ethnicity, marital status, insurance, admission type, temperature, heart rate, MAP, respiratory rate, SPO_2_, Glucose, WBC, SCr, hemoglobin, platelets, SAPSII score, SOFA score, PaO_2_/FiO_2_, ALT, AST, ALP, bilirubin, acetaminophen route, infection site, vasopressin use, CRRT use, aspirin use, statin use, myocardial infarct, congestive heart failure, dementia, cerebrovascular disease, chronic pulmonary disease, Mild liver disease, renal disease). HR, hazard ratio; CI, confidence interval; ICU, intensive care unit; IQR, interquartile range.

After using PSM, we found consistent results with the PSM group, demonstrating that administering acetaminophen was connected to lower in-hospital mortality (HR 0.443; 95% CI 0.371–0.53; *p* < 0.001). Also, the acetaminophen administration was linked to lower 30-day mortality (HR 0.497; 95% CI 0.424–0.583; *p* < 0.001). It further led to shorter hospital [8.4 (5.0, 14.8) vs. 9.0 (5.1, 16.0), *p* < 0.001] and ICU stays [2.8 (1.5, 6.0) vs. 3.1 (1.7, 6.5); *p* < 0.05] (Table 2).

### 3.3 Subgroup analysis

**FIGURE 3:**
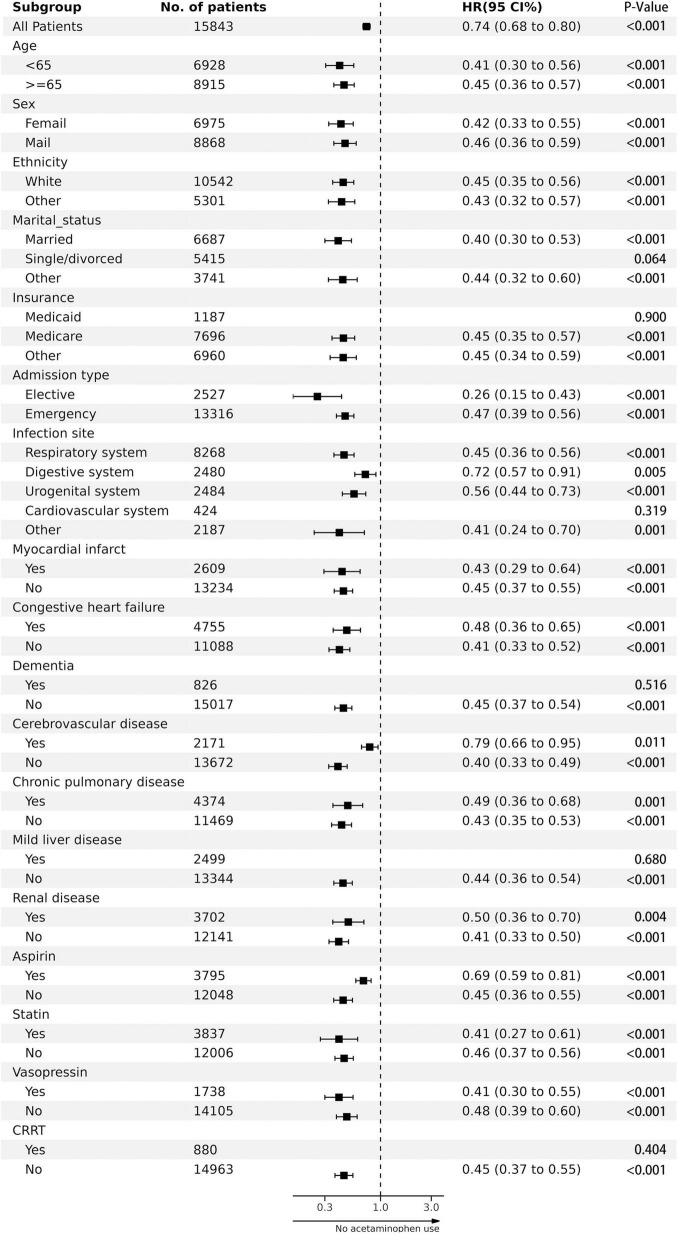
The association between acetaminophen adminstration and in-hospital mortality in subgroups.

Figure 3’s study suggests that the lower in-hospital mortality rate associated with acetaminophen is linked to various factors like age, sex, ethnicity, and admission type. It also correlates with the presence of certain conditions, such as myocardial infarction, congestive heart failure, cerebrovascular disease, chronic pulmonary disease, and renal disease. Acetaminophen usage in the form of aspirin, statins, and vasopressors also seemed to influence the lower mortality rate.

Furthermore, other elements such as marital status (married or other), insurance type (medicare or other), infection site (respiratory, digestive or urogenital system, among others), lack of dementia and mild liver disease, and the non-use of CRRT also showed a correlation with lower in-hospital mortality rates.

## 4 Discussion

Our research reveals that administering acetaminophen has a link to a lower in-hospital mortality in patients with critical sepsis. The outcomes from this group also imply that acetaminophen can potentially lower 30-day mortality from sepsis and expedite hospital discharge and ICU discharge. These results maintain their strength even following adjustments for risk factors and the application of PSM for comparison. Our findings endorse the use of acetaminophen in sepsis treatment, suggesting a promising therapeutic option for clinical practice and inviting further research in this area.

Our study aligns with early clinical trials suggesting that acetaminophen could improve lipid peroxidation and kidney function in septic patients (37). An earlier observational study found a correlation between acetaminophen use and decreased in-hospital deaths in critically unwell septic patients. This implies a protective effect by reducing oxidative damage caused by cell-free hemoglobin (38). Moreover, some researchers have noted that giving acetaminophen within the first 24 h of ICU admission can lessen oxidative damage and enhance kidney function in severely septic adults. This is particularly true when cell-free hemoglobin is detectable in the blood plasma (14) .

A controlled study examined 700 patients with fever, indicating that the early use of acetaminophen to treat potential infection neither impacted the length of ICU stays nor affected survival rates at the 28-day and 90-day milestones (20). However, our research, unlike the study mentioned above, focused specifically on sepsis patients. This approach provided a clearer view of acetaminophen’s role in sepsis management. The study concluded that administrating acetaminophen to sepsis patients can shorten time spent in the ICU and lower short-term mortality rates.

In a cohort study of 606 sepsis patients, researchers observed a notable link between the use of acetaminophen and increased mortality among those who had a fever (39). What makes our study unique is our wider scope. We did not focus solely on fever patients but on various forms of sepsis for all reasons. We utilized the Sepsis-3 criteria to define sepsis, incorporating a more diverse range of subjects for a complete depiction of the sepsis patient population. Unlike past results, our study indicates a significant correlation between the use of acetaminophen and a reduction in mortality.

In a past study, researchers examined 46 pediatric sepsis patients aged 7 to 18. They found no link between acetaminophen use and increasing organ dysfunction or mortality rates (40). Unlike this study, our research primarily targets adult patients due to the higher occurrence of sepsis in adults versus children. Moreover, our study involves a larger sample size, rendering our results more clinically relevant.

Research indicates that the common anti-inflammatory and antioxidant medication, acetaminophen, shows noteworthy potential in treating sepsis (13). Essentially, acetaminophen might provide a safeguard by minimizing oxidative damage instigated by cell-free hemoglobin. It can connect to ferrous iron (Fe^4 +^) in cell-free hemoglobin at clinically relevant doses, altering it to a less reactive ferric iron (Fe^3 +^) (38, 41, 42). In lab tests, some studies have suggested that acetaminophen eases sepsis-induced cognitive impairment by reducing iron-induced cell death via the GPX4 and FSP1 signal pathways (43, 44). Other research hints that it reduces the effect of endotoxins on pulmonary circulation in sedated pigs, which could be crucial in severe systematic inflammation (45). Moreover, the CYP3A5 gene has been proposed as a potential significant biomarker for acetaminophen metabolism. Understanding certain genotypes linked to acetaminophen reactions could lead to more tailored treatment methods for handling sepsis and septic shock (46). Looking forward, more research is required to understand the molecular mechanics and biochemical responses of acetaminophen in sepsis treatments, gathering evidence for its positive effects in clinical contexts.

While this study offers significant findings, it is not without constraints. First, as with all retrospective analyses, potential confounding elements like a patient’s underlying medical conditions, lifestyle, and personal habits could influence results. We minimized these influences by adjusting for possible confounders using PSM. Second, our study only considered drugs like acetaminophen, disregarding other treatment interventions. The multi-faceted nature of sepsis treatment warrants further research comparing different methods. Third, our analysis only included patients treated with acetaminophen within 48 h of admission, leaving the effects of delayed use uncertain. More research is needed in this area. Fourth, the MIMIC-IV database, which does not record death causes, has some data limitations, hampering our ability to conduct a competing risk analysis. Lastly, our study is single-center, necessitating validation through multicenter trials. Given these constraints, future research should explore acetaminophen’s exact role in sepsis treatment and provide more comprehensive analyses to confirm our findings.

## 5 Conclusion

Acetaminophen use may be linked to lower short-term mortality in critically ill septic patients, according to our study’s findings. This implies that the careful use of acetaminophen can benefit such patients. However, more comprehensive studies, like multicenter randomized controlled trials, are needed to validate and confirm this correlation further.

## Data availability statement

The raw data supporting the conclusions of this article will be made available by the authors, without undue reservation.

## Ethics statement

The studies involving humans were approved by the Massachusetts Institute of Technology and Beth Israel Deaconess Medical Center. The studies were conducted in accordance with the local legislation and institutional requirements. The participants provided their written informed consent to participate in this study. Written informed consent was obtained from the individual(s) for the publication of any potentially identifiable images or data included in this article.

## Author contributions

SS: Conceptualization, Data curation, Writing – original draft, Writing – review & editing. HL: Data curation, Writing – original draft. QL: Conceptualization, Writing – review & editing, Writing – original draft. YY: Data curation, Writing – original draft. XC: Data curation, Writing – original draft. BZ: Data curation, Writing – original draft.
